# Todesursachenstatistik – wie Fehlinterpretationen von Mortalitätsdaten vermieden werden

**DOI:** 10.1007/s00103-024-03986-3

**Published:** 2024-12-04

**Authors:** Susanne Stolpe, Bernd Kowall

**Affiliations:** Institut für medizinische Informatik, Biometrie und Epidemiologie, Universitätsklinikum Essen, Hufelandstr. 55, 45147 Essen, Deutschland

**Keywords:** Unikausale Todesursachenstatistik, Grundleiden, Validität vergleichender Schlussfolgerungen aus Mortalitätsdaten, Nichtinformative Todesursachen, Letalität, Unicausal cause-of-death statistic, Underlying cause of death, Validity of comparative conclusions from cause-of-death statistics, Ill-defined causes of death, Lethality

## Abstract

Nationale Mortalitätsregister sind eine wichtige Datenquelle für das Monitoring der Bevölkerungsgesundheit. Aus Analysen insbesondere der kardiovaskulären Mortalität und speziell der Mortalität an koronarer Herzkrankheit werden Rückschlüsse auf die Qualität der Gesundheitsversorgung und Prävention gezogen. Um krankheitsspezifische Mortalitätsunterschiede zwischen Ländern und Veränderungen über die Zeit interpretieren zu können, ist es jedoch notwendig, die Grundlagen der unikausalen Todesursachenstatistik und die damit verbundenen Einschränkungen bei vergleichenden Aussagen zu kennen.

Schlussfolgerungen aus krankheitsspezifischen Mortalitätsdaten können wegen der sehr subjektiven Auswahl von Erkrankungen, die nach einer Leichenschau im Totenschein erfasst werden, problematisch sein. Unkenntnis der Leichenschauenden darüber, welche verschiedenen „Rollen“ einzelne, zum Zeitpunkt des Todes bekannte Erkrankungen innerhalb der zum Tode führenden Kausalkette einnehmen, kann zu unzureichend ausgefüllten Totenscheinen als Datengrundlage der Mortalitätsstatistik führen. Vergleiche krankheitsspezifischer Mortalitätsraten werden so durch verschiedene Anteile nichtinformativer, u. a. auch unbekannter Todesursachen und landesspezifische Präferenzen bei der Eintragung von Erkrankungen im Totenschein erschwert – insbesondere bei Multimorbidität. Die Morbidität einer Bevölkerung wird daher durch Mortalitätsraten nur eingeschränkt widergespiegelt. Begriffliche Unklarheiten in Bezug auf die Konzepte Letalität und Mortalität bei der Beschreibung von Mortalitätsraten können ebenfalls zu fehlerhaften Schlussfolgerungen führen.

Schulungen des ärztlichen Personals zum Ausfüllen eines Totenscheins und die geplante elektronische Todesbescheinigung könnten die Datengrundlage verbessern. Unabhängig davon verbessert die Kenntnis möglicher Fallstricke bei der Nutzung von Mortalitätsdaten die Qualität der Gesundheitsberichterstattung.

## Abstract (en)

National mortality registers provide important data for monitoring population health. Analyses of cardiovascular mortality in particular—and especially mortality from coronary heart disease—are frequently the basis for conclusions about the quality of healthcare and prevention. To be able to interpret disease-specific mortality differences between countries and changes in mortality over time, it is necessary to know the basics of monocausal cause-of-death statistics and the associated limitations in comparative statements.

Conclusions from disease-specific mortality data can be problematic due to the highly subjective selection of diseases that are entered on a death certificate after a post-mortem examination. In death certification, unawareness of the different “roles” of specific diseases—known at the time of death—within the causal chain leading to death can result in incomplete death certificates as a data basis for mortality statistics. Comparisons of disease-specific mortality rates are difficult due to different proportions of non-informative—including unknown—causes of death and due to country-specific preferences for which diseases are recorded on a death certificate—especially in the prevalence of multimorbidity. A population’s morbidity is therefore only reflected to a limited extent by mortality rates. Conceptual ambiguities with regard to the concepts of lethality and mortality when describing mortality rates can also lead to erroneous conclusions.

Training of medical staff on how to complete a death certificate and the introduction of an electronic death certificate can improve the quality of mortality data. Irrespective of this, knowing potential pitfalls when analyzing mortality data will improve the quality of health reporting.

## Hintergrund

Die nationalen Todesursachenregister sind eine der wichtigsten Quellen für Informationen zur Morbidität einer Bevölkerung und deren Veränderung über die Zeit. Vergleiche von ursachenspezifischen Mortalitätsraten dienen dazu, diejenigen Erkrankungen zu identifizieren, die für das Mortalitätsgeschehen einer Bevölkerung von besonderer Bedeutung sind und deshalb im Fokus der Versorgung stehen sollten. Zusätzlich können niedrigere Mortalitätsraten für einzelne Erkrankungen in anderen Ländern darauf hinweisen, dass das Potenzial zur Vermeidung der Erkrankung und dadurch verursachter Todesfälle noch nicht ausgeschöpft ist.

Gleichzeitig werden aus Vergleichen von Mortalitätsraten Rückschlüsse auf die Qualität der gesundheitlichen Versorgung einer Erkrankung gezogen. Dies insbesondere bei Erkrankungen, bei denen eine zeitnahe leitlinienkonforme Intervention des Gesundheitssystems die Letalität deutlich verringert, wie z. B. bei akutem Herzinfarkt oder Schlaganfall. Hier wird impliziert, dass bei besserer krankheitsspezifischer Versorgung weniger Betroffene an ihrer Erkrankung versterben, das heißt, dass die Letalität sinkt und daraus folgend auch niedrigere Mortalitätsraten zu verzeichnen sind.

Vor einer Interpretation der Todesursachenstatistik ist es jedoch unbedingt notwendig, die Herkunft der Daten und die Art ihres Entstehens zu kennen. Wie auch Ben Jones in seinem Buch *Avoiding data pitfalls* beschreibt, müssen die Auswertenden wissen, was die Daten abbilden – und noch wichtiger, was sie *nicht* abbilden, um Fallstricke („pitfalls“) bei der Interpretation zu vermeiden [1].

Die Mortalitätsstatistik bildet die Realität bezogen auf das Alter und Geschlecht aller Verstorbenen ab. Die Annahme, dass die krankheitsspezifische Morbidität einer Bevölkerung ebenso gut abgebildet wird, stimmt jedoch unter anderem wegen häufiger Multimorbidität nur eingeschränkt [2].

In diesem Diskussionsbeitrag werden zunächst die Grundlagen der unikausalen Todesursachenstatistik gemäß den Regeln der Weltgesundheitsorganisation (WHO) dargestellt, insbesondere welche Arten von Todesursachen unterschieden werden und welche Probleme bei der Selektion des Grundleidens existieren. Anschließend wird dargestellt, welchen Einfluss unterschiedliche Anteile nichtinformativer Todesursachen, Multimorbidität und nationale oder zeitlich veränderliche Priorisierungen von Todesursachen auf die Aussagefähigkeit von Todesursachenstatistiken haben. Eine Abgrenzung von Mortalität und Letalität soll schließlich zusätzlich dazu beitragen, Fallstricke bei der Analyse und Interpretation der Daten der Todesursachenstatistik zu vermeiden.

## Grundlage einer unikausalen Mortalitätsstatistik

Die Todesursachenstatistik des statistischen Bundesamtes in Deutschland wird *unikausal* zur Verfügung gestellt: Von häufig mehreren prävalenten Erkrankungen zum Zeitpunkt des Todes [3], die im besten Falle vollständig auf einem Totenschein eingetragen wurden, wird nur eine Erkrankung oder externe Ursache als Todesursache gespeichert.

Um eine internationale Vergleichbarkeit der Todesursachenstatistiken zu ermöglichen, wurde von der WHO 1948 festgelegt, dass nur das Grundleiden als Todesursache gemäß der internationalen statistischen Klassifikation der Krankheiten und verwandter Gesundheitsprobleme (ICD) kodiert dokumentiert wird [4]. Als *Grundleiden* wird diejenige Erkrankung oder externe Ursache bezeichnet, die am Beginn einer Kette von Folgeerkrankungen und Zuständen stand, die letztendlich zum Tode führten: „the disease or injury which initiated the train of morbid events leading directly to death, or the circumstances of the accident or violence which produced the fatal injury“ [4].

**Abb. 1:**
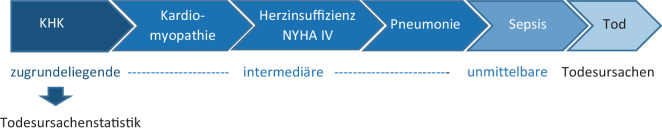
Kausalkette von zum Tode führenden Erkrankungen und Systematik der Todesursachen (Quelle: eigene Abbildung); *KHK* koronare Herzkrankheit, *NYHA* New York Heart Association = Schema zur Einteilung von Herzkrankheiten nach Schweregrad

Zum Zeitpunkt des Todes prävalente Erkrankungen und Zustände, die durch das Grundleiden oder durch Folgekrankheiten ausgelöst wurden, sind ebenfalls Todesursachen. Sie werden aber in Abgrenzung zum Grundleiden als *intermediäre Todesursachen* bezeichnet. Dies kann ein Myokardinfarkt nach Atherosklerose sein, Lebermetastasen ausgelöst durch Magenkrebs, eine Sepsis nach Harnwegsinfekt oder, wie in Abb. 1 dargestellt, eine Herzinsuffizienz ausgelöst durch eine Kardiomyopathie oder koronare Herzerkrankung (KHK). Erkrankungen oder Zustände, die am Ende der Kausalkette stehen und dem Tod unmittelbar vorausgingen, werden als *unmittelbare Todesursachen* bezeichnet. Häufig werden Endzustände wie Herzstillstand, respiratorische Insuffizienz oder Multiorganversagen als unmittelbare Todesursache im Totenschein aufgeführt.

Intermediäre und unmittelbare Todesursachen beschreiben *nicht* das Grundleiden und werden somit *nicht* in der Todesursachenstatistik gespeichert – sofern andere Erkrankungen im Totenschein genannt sind, die sich als Grundleiden qualifizieren. Eine unikausale Todesursachenstatistik ist als Quelle zur Auskunft über die Mortalität an möglichen Grundleiden angelegt, d. h., um die Erkrankungen zu dokumentieren, die in einer Population hätten vermieden oder besser behandelt werden müssen, um Todesfälle letztendlich zu vermeiden oder zu verzögern. Welche Erkrankung oder Kombination zweier Erkrankungen (z. B. hypertensive Nierenkrankheit) aufgrund der Eintragungen in beiden Teilen des Totenscheins im Rahmen der automatisierten oder manuellen Kodierung als Grundleiden in der Todesursachenstatistik registriert wird, entscheidet das im Totenschein eingetragene Ergebnis der Leichenschau [5].

### Regeln im Rahmen der Dokumentation von Todesursachen – Leichenschau

Sowohl das Vorgehen bei einer Leichenschau als auch das Ausfüllen des Totenscheins und die Selektion des Grundleidens sind geregelt [6, 7] und eine möglichst gute Durchführung im allgemeinen Interesse [8]. Es ist jedoch anzunehmen, dass allein das obligatorische Entkleiden der verstorbenen Person aus Gründen wie Zeitmangel, Pietät gegenüber anwesenden Angehörigen, fehlender personeller Unterstützung beim Bewegen der Leiche oder bereits eingesetzter Leichenstarre nicht immer vorgenommen wird. Eine Leitlinie der Arbeitsgemeinschaft der Wissenschaftlichen Medizinischen Fachgesellschaften e. V. (AWMF) beschreibt die Leichenschau als einen „Akt hoher ärztlicher Verantwortung“, denn „mit der Ausstellung der Todesbescheinigung werden die Weichen gestellt, ob die Leiche ohne weitere Kontrolle bestattet wird oder ob weitere Ermittlungen im Hinblick auf einen nicht natürlichen Tod erforderlich sind“ [6]. Auf den letzten Punkt fokussieren sich Medienberichte^1^ über die Folgen einer nicht sachgerechten Leichenschau. Die Zahl der unentdeckten Tötungsfälle wurde auf jährlich 1200 [9] und 2000 [10] geschätzt. Beispielsweise wurde in Bremen seit 2017 nur ein Tötungsdelikt durch eine qualifizierte Leichenschau in der Rechtsmedizin aufgedeckt, bei insgesamt 56.808 Verstorbenen [11].

Die AWMF-Leitlinie erwähnt einen weiteren, aus Sicht von Public Health wichtigeren Aspekt: Das sorgfältige Ausfüllen eines Totenscheins ist zwar eine Handlung an einer verstorbenen Person, aber immer auch eine ärztliche Dienstleistung für alle noch Lebenden. Diese können von Interventionen des Gesundheitswesens oder besseren Versorgungsroutinen profitieren, die aufgrund von Todesursachenanalysen initiiert oder priorisiert wurden.

### Regeln zum Ausfüllen des Totenscheins und zur Selektion des Grundleidens

Vollständig und sachgerecht ausgefüllte Totenscheine sind die Grundlage für Mortalitätsanalysen, die zur Verbesserung der Gesundheitsversorgung beitragen können. Das WHO-Regelwerk, nach dem das Grundleiden aus möglicherweise mehreren im Totenschein aufgeführten Erkrankungen ausgewählt und ICD-kodiert wird, ist sehr ausführlich und wird regelmäßig aktualisiert [7]. Diese Regeln sollten den Leichenschauenden zumindest in Grundzügen bekannt sein. Davon abweichende Einträge können bei der Bearbeitung des Totenscheins durch Kodierfachkräfte oder durch die Software IRIS/Muse in statistischen Ämtern zur Selektion eines anderen als des intendierten Grundleidens führen.

**Abb. 2:**
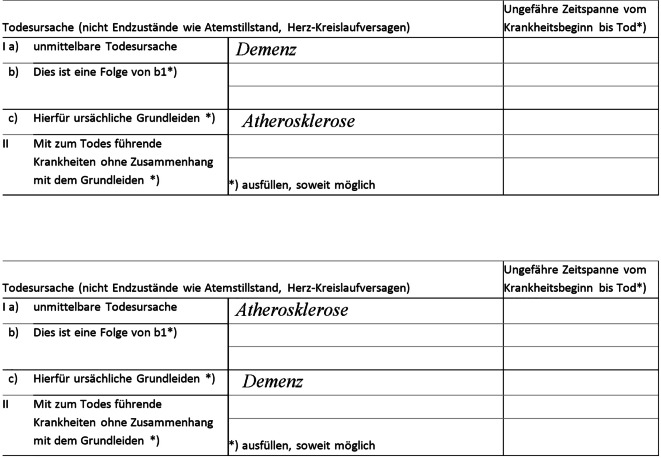
Beispiel für das Ausfüllen eines Totenscheins mit gleichen Erkrankungen, aber unterschiedlicher zugrunde liegender Todesursache als Ergebnis der anschließenden manuellen oder automatisierten Kodierung (vaskuläre Demenz (oben) und Atherosklerose (unten); Quelle: [7])

Bei der Ermittlung des Grundleidens können laut Regelwerk nur Erkrankungen berücksichtigt werden, die im Totenschein eingetragen sind. Den Leichenschauenden muss daher bekannt sein, dass einzelne Erkrankungen innerhalb der zum Tode führenden Kausalkette aus Sicht der Mortalitätsstatistik unterschiedliche „Rollen“ einnehmen: Sie können als zugrunde liegende (= Grundleiden), intermediäre oder unmittelbare Todesursache betrachtet werden. Zudem muss bekannt sein, wie diese „Rollen“ durch die Positionierung einer Erkrankung auf den Zeilen des Totenscheins wiedergegeben werden. Obwohl die gleichen Erkrankungen im Totenschein eingetragen sind, würde in Abb. 2 im ersten Beispiel eine vaskuläre Demenz, im zweiten Beispiel eine Atherosklerose als Todesursache erfasst werden [7].

### Nichtinformative Todesursachen („ill-defined causes of death“)

Eine unikausale Todesursachenstatistik dokumentiert nur das Grundleiden. Das bedeutet, dass nur intermediär auftretende Erkrankungen oder Endzustände nicht in der Todesursachenstatistik vorkommen dürften, wie z. B. die intermediären Todesursachen Herzinsuffizienz (I50), Sepsis (A41) und nicht näher bezeichnete Pneumonie (J18.9) ≙ 90 % aller Sterbefälle mit Pneumonie als kodiertem Grundleiden (16.171 von 18.071 Sterbefällen an J18 im Jahr 2019 (gbe-bund.de)) oder Endzustände wie Herzstillstand (I46). Dies gilt auch für Sterbefälle mit einem Symptom als Todesursache, wie essenzielle Hypertonie (I10). Todesursachen aus dem ICD-10-Kapitel XVIII („Symptome und abnorme klinische und Laborbefunde, die anderenorts nicht klassifiziert sind“, R00-R94, R96-R99) sind ebenfalls kein Grundleiden. Trotzdem wurde 2019 in Deutschland in 35.000 Fällen als Todesursache „Sonstige ungenau oder nicht näher bezeichnete Todesursache“ (R99), „Tod ohne Anwesenheit einer anderen Person“ (R98) oder „Senilität“ (R54) in der Todesursachenstatistik registriert, da nach der Leichenschau nur eine dieser Angaben im Totenschein gemacht wurde (gbe-bund.de).

Die Dokumentation unbekannter, unmittelbarer oder intermediärer Todesursachen in einer unikausalen Todesursachenstatistik ist problematisch, da so die eigentliche, für den Tod verantwortliche Grunderkrankung verborgen bleibt. Im Sinne von Public Health kann nicht erkannt werden, welche Erkrankungen für die Mortalität einer Bevölkerung im Besonderen verantwortlich sind. Dies ist insbesondere bei Endzuständen als Todesursachen oder unbekannten Todesursachen (ICD-10 R00-R94, R96-R99) leicht erkennbar. Endzustände und Symptome, nur intermediär auftretende und unbekannte Todesursachen werden von der WHO als „ill-defined causes of death“ oder „garbage codes“ bezeichnet [12]. In Deutschland ist die Benennung „nichtinformative Todesursache“ geläufiger.

Je kleiner der Anteil dieser Todesursachen in einer Mortalitätsstatistik, desto besser wird deren Qualität durch die WHO beurteilt. Nach Daten der WHO lag der Anteil nichtinformativer Todesursachen in Deutschland zwischen 1998 und 2018 zwischen 14 % und 17 % und damit deutlich höher als in Finnland (3 %), Irland, Ungarn und Estland (7 %) oder im Vereinigten Königreich (8 %; [13]). In diesen Ländern liegen die Mortalitätsraten z. B. für Herzinsuffizienz oder unbekannte Todesursachen nahe null, was auch darauf zurückzuführen ist, dass zumindest in Finnland und Großbritannien speziell geschultes Personal für die Leichenschau zuständig ist.

In Deutschland dagegen werden in der Gesundheitsberichterstattung Mortalitätsraten und deren Veränderung für nichtinformative Todesursachen, wie Herzinsuffizienz oder Sepsis, interpretiert. So ergab eine Studie des Kompetenznetzes Sepsis (SepNet), dass in Deutschland etwa 162 Menschen täglich an Sepsis sterben. Dies wird mit den Zahlen der offiziellen Todesursachenstatistik kontrastiert, nach der nur 16,7 Sepsistote täglich erwartet werden können. Die Sepsis sei daher ein „unbekannter Killer“. Da eine Sepsis intermediär als Folge einer Infektion auftritt und unmittelbare Todesursache ist, müsste korrekterweise die Infektion als Todesursache registriert werden. Die Häufigkeit von Todesfällen mit Sepsis als unmittelbarer Todesursache kann nicht über die Todesursachenstatistik geschätzt werden [14].

## Folge unterschiedlicher Anteile nichtinformativer Todesursachen in der Todesursachenstatistik

Je unterschiedlicher der Anteil nichtinformativer Todesursachen in Todesursachenstatistiken, desto eingeschränkter ist die Vergleichbarkeit der Mortalitätsraten. Allein zwischen den deutschen Bundesländern existieren deutliche Unterschiede in den jeweiligen Anteilen: 2019 betraf dies etwa 6 % aller Todesfälle in Sachsen im Vergleich zu 12 % in NRW (gbe-bund.de). Auch die Einführung automatisierter Todesursachenkodierung mit der Software IRIS/Muse [15] hat die Anteile nichtinformativer Todesursachen nur wenig und nur für kurze Zeit verringert [16].

**Abb. 3:**
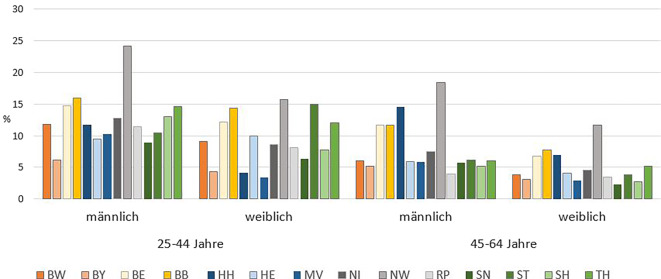
Anteil unbekannter Todesursachen (ICD-10 R00-R94, R96-R99) in Bundesländern nach Alter und Geschlecht 2019 (Quelle: eigene Abbildung). *BW* Baden-Württemberg, *BY* Bayern, *BE* Berlin, *BB* Brandenburg, *HH* Hamburg, HE Hessen, *MW* Mecklenburg-Vorpommern, *NI* Niedersachsen, *NW* Nordrhein-Westfalen, *RP* Rheinland-Pfalz, *SN* Sachsen, *ST* Sachsen-Anhalt, *SH* Schleswig-Holstein, *TH* Thüringen. Saarland und Bremen wurden wegen besserer Übersichtlichkeit nicht mit dargestellt

Obendrein ist der Anteil nichtinformativer Todesursachen geschlechts- und altersabhängig und verändert sich über die Zeit. So betrug 2019 der Anteil nichtinformativer Todesursachen (R00–R94, R96–R99) in Deutschland bei den 25- bis 44-Jährigen 14 % bei Männern und 10 % bei Frauen, bei den 45- bis 64-Jährigen 9 % bei Männern und 6 % bei Frauen und bei Verstorbenen im Alter von > 64 Jahren etwa 3 % jeweils bei Männern und Frauen (Abb. 3; gbe-bund.de). Für NRW waren 2019 zu 3501 Todesfällen von Männern im Alter zwischen 25 und 64 Jahren keine Angaben zur Todesursache verfügbar (19 %), im Gegensatz zu 5 % in Bayern (*N* = 588). Dabei erscheint aus Sicht von Public Health der hohe Anteil unbekannter Todesursachen in jüngeren Altersgruppen problematisch, da keine Interventionsmöglichkeiten abgeleitet werden können, um künftig vorzeitige Todesfälle und verlorene Lebensjahre zu vermeiden.

Je höher der Anteil nichtinformativer Todesursachen und je unterschiedlicher dieser Anteil in Vergleichsregionen, zwischen Geschlechtern, Altersgruppen oder über die Zeit, desto weniger valide können Schlussfolgerungen aus (geschlechts- und altersspezifischen) Vergleichen von Mortalitätsraten gezogen werden. In den ostdeutschen Bundesländern ist z. B. die Mortalitätsrate für akuten Myokardinfarkt höher als in den meisten westdeutschen Bundesländern [17]. Dies könnte an Unterschieden in der Gesundheitsversorgung liegen, aber auch dadurch mitverursacht sein, dass in diesen Bundesländern mehr informative Todesursachen und damit potenziell auch mehr Myokardinfarkte registriert werden [18].

**Abb. 4:**
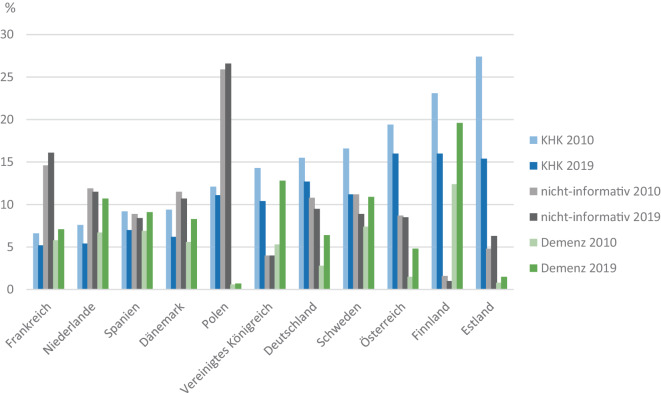
Anteil von koronarer Herzkrankheit (KHK), Demenz und nichtinformativen Todesursachen an allen Todesfällen in verschiedenen europäischen Ländern 2010 und 2019 (Quelle: eigene Abbildung)

Ähnliches gilt auch für den Vergleich zwischen Ländern. In Finnland wurde 2019 nur 1 % aller Todesfälle mit einer nichtinformativen kardiovaskulären oder unbekannten Todesursache registriert, im Vergleich zu 10 % in Deutschland und 16 % in Frankreich (Abb. 4). Zur besseren Vergleichbarkeit von Mortalitätsdaten aus unterschiedlichen Regionen und Jahren werden in den Berichten der Global Burden of Disease Study (GBD) nichtinformative Todesursachen umkodiert [19, 20]. So kann z. B. bei einem Todesfall an Herzinsuffizienz zu etwa 77 % eine KHK (I20–I25) als Auslöser angenommen werden [12]. Gemäß den genaueren alters- und geschlechtsspezifisch geschätzten Wahrscheinlichkeiten wird bei den entsprechenden Anteilen der Todesfälle die Todesursache „Herzinsuffizienz“ zu „KHK“ umkodiert.

## Folgen der Multimorbidität für die Todesursachenstatistik

**Abb. 5:**
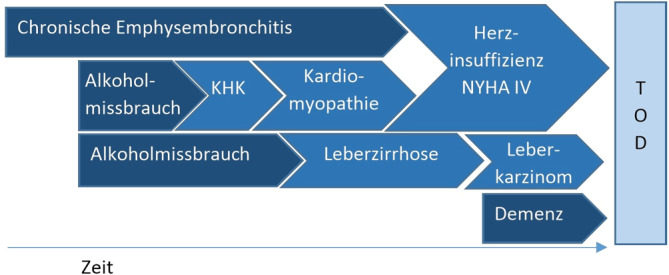
Beispiel von Multimorbidität bei einem Todesfall mit mehreren möglichen Grundleiden und Kausalketten (Quelle: eigene Abbildung). *KHK* koronare Herzkrankheit, *NYHA* New York Heart Association = Schema zur Einteilung von Herzkrankheiten nach Schweregrad

Welche von mehreren im Todesfall prävalenten Erkrankungen als Grundleiden auf einem Totenschein eingetragen wird, hängt u. a. auch vom Sterbeort, der Auffindesituation, vom Alter und weiteren verfügbaren Informationen über die verstorbene Person ab. So ist es beispielsweise vorstellbar, dass eine Person mit der in Abb. 5 beschriebenen Morbidität im Hospiz verstirbt und als Grundleiden „Leberkarzinom“ ausgewählt wird. Bei Tod nach Krankenhausaufnahme mit der Indikation „Herzinsuffizienz“ könnte bei der Leichenschau die gemäß Patientenakte zuletzt behandelte kardiale Ursache „KHK“ oder „Herzinsuffizienz“ mehr im Fokus stehen. Ein über lange Zeit behandelnder Hausarzt hätte bei einer Leichenschau am Wohnort möglicherweise eher Kenntnis vom Alkoholmissbrauch und bei einem Tod im Pflegeheim wäre ggf. die Demenz das zentrale Thema für die Betreuenden. Je nach Alter einer verstorbenen Person und Sorgfalt bei der Leichenschau könnten aber auch Einträge wie „Senilität“ oder „Herzstillstand“ im Totenschein vorkommen.

**Abb. 6:**
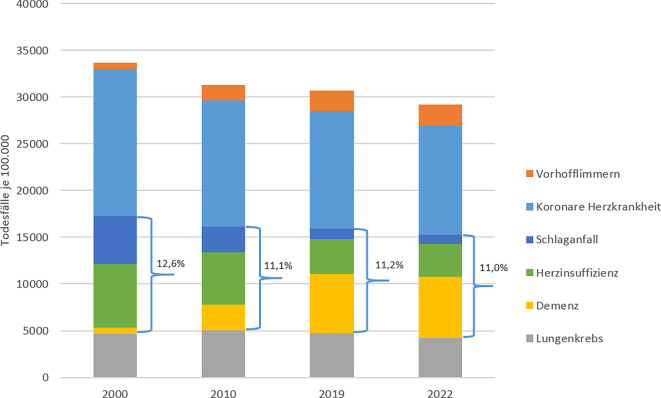
Todesfälle je 100.000 Personen an ausgewählten Todesursachen 2000–2022 in Deutschland und gemeinsamer Anteil der Todesursachen Schlaganfall, Herzinsuffizienz und Demenz an allen Todesfällen (Quelle: eigene Abbildung)

Seit 2011 soll Demenz nach den WHO-Regeln vorrangig vor anderen im Totenschein aufgeführten Erkrankungen, die sich als Grundleiden qualifizieren (z. B. Schlaganfall), als Grundleiden zur Kodierung ausgewählt werden. Folge dieser Regeländerung ist ein starker Anstieg der Mortalitätsrate für Demenz in vielen europäischen Ländern [2, 21]. In Deutschland war die „nicht näher bezeichnete Demenz“ (F03) im Jahr 2010 mit 15.591 Sterbefällen noch auf Platz 12 der Todesursachen, im Jahr 2022 mit 53.323 Todesfällen auf Platz 2 (gbe-bund.de). Personen mit Demenz haben häufig Komorbiditäten [22]. Bei steigender Häufigkeit der Todesursache Demenz müssen die Mortalitätsraten anderer Erkrankungen zurückgehen, da ja die Gesamtzahl der Sterbefälle nicht in gleichem Umfang zunimmt. Sinkende Sterbefallzahlen sind vor allem für kardiovaskuläre Erkrankungen des höheren Alters wie Herzinsuffizienz und Schlaganfall zu verzeichnen (Abb. 6).

Jedoch führen nicht nur Regeländerungen zu Veränderungen der Todesursachenhäufigkeit. Vorhofflimmern und -flattern (I48) wird seit dem Jahr 2000 nicht nur in Deutschland immer häufiger als Grundleiden registriert, da auch in Deutschland der Anteil hochaltriger Menschen kontinuierlich ansteigt, bei denen diese Erkrankung überhaupt auftreten, diagnostiziert und behandelt werden kann [17]. In einem Report zur kardiovaskulären Mortalität wurde 2015 explizit die ansteigende Mortalität für Vorhofflimmern und -flattern mit einem steigenden Bewusstsein für diese Erkrankung, häufigerem Screening und besserer Behandlung anderer, assoziierter Erkrankungen wie KHK, Schlaganfall und Diabetes in Zusammenhang gebracht [23]. Im GBD-Report des gleichen Jahres wird auch die ansteigende Mortalität für Demenz mit der erhöhten Bereitschaft, diese Erkrankungen als Todesursache einzutragen, begründet [24]. Diese Aussagen unterstreichen die Subjektivität der Entscheidung, welche Erkrankung im Totenschein eingetragen wird. Abb. 6 zeigt, dass in Deutschland der gemeinsame Anteil der Todesursachen Demenz (F01, F03, G30), Herzinsuffizienz (I50) und Schlaganfall (I64) an allen Todesursachen seit 2010 in etwa gleichgeblieben ist, während sich die Anteile dieser Todesursachen im Einzelnen veränderten.

Rückläufige Mortalitätsraten für Herzinsuffizienz, Schlaganfall und insbesondere auch für KHK sind daher auch immer eine Folge sich verändernder Mortalitätsraten anderer Erkrankungen und lassen anders als oft publiziert wenig Rückschlüsse auf eine Verbesserung der Versorgungsqualität zu [17]. Die in Deutschland tendenziell rückläufige Häufigkeit von Herzinsuffizienz als Todesursache könnte auch die Folge einer verbesserten Qualität der Todesursachenstatistik durch häufigere Selektion der Todesursache Demenz sein – möglicherweise als Folge der Einführung der automatisierten Kodierung [16].

Der Anteil von Lungenkrebs als Todesursache dagegen blieb seit 2000 in etwa gleich (Abb. 6). Bei Todesfällen aufgrund von Krebserkrankungen oder auch Unfällen scheint es im Rahmen einer Leichenschau zum einen eher möglich, ein eindeutiges Grundleiden zu identifizieren, zum anderen ist das Bewusstsein für die Relevanz dieser Todesursachen vielleicht geringeren Schwankungen bzw. Änderungen unterworfen.

## Landesspezifische Prioritäten bei der Eintragung von Todesursachen im Totenschein

Nicht nur innerhalb Deutschlands, sondern auch zwischen europäischen Ländern gibt es große Unterschiede im Anteil einzelner Todesursachen an allen Todesfällen. Insbesondere die Todesursachen „KHK“, „hypertensive Herzkrankheit“, „Kardiomyopathie“ und „Demenz“ wie auch nichtinformative Todesursachen werden in sehr unterschiedlichem Maß als Grundleiden im Totenschein eingetragen [2]. So ging beispielsweise der Anteil von KHK an allen Todesursachen von 2010 bis 2019 deutlich zurück und betrug dann in Frankreich 5,2 % (2010: 6,6 %), in Deutschland 12,7 % (15,5 %) und in Estland 15,4 % (27,4 %; Abb. 4). Die altersstandardisierten Mortalitätsraten für KHK lagen 2019 zwischen 18,2/100.000 in Frankreich und 62,2 in Estland (Deutschland: 44,7). Diese Unterschiede wie auch das Ausmaß der Änderung seit 2010 sind zu groß, als dass sie nur über die Morbidität der jeweiligen Bevölkerungen oder mit Unterschieden in der Gesundheitsversorgung erklärt werden könnten.

Für die Todesursache KHK, aber auch für die kardiovaskulären Todesursachen (I00–I99) insgesamt, sind diese schwer zu erklärenden Unterschiede zwischen Ländern und über die Zeit besonders groß. Gerade die kardiovaskuläre Mortalität und insbesondere die KHK-Mortalität werden international als Benchmark für die (Verbesserung der) Qualität der Gesundheitsversorgung gesehen. Es ist aber anzunehmen, dass Schlussfolgerungen zum Rückgang der Mortalität von KHK bzw. der kardiovaskulären Mortalität zumindest seit den 1990er-Jahren aufgrund von Fortschritten in Prävention und Therapie [17, 25–29] auf einer eingeschränkt validen Basis gezogen wurden.

Ganz allgemein kann nicht davon ausgegangen werden, dass für eine Person gleichen Geschlechts und Alters mit gleicher Komorbidität im Todesfall in unterschiedlichen Ländern (oder auch in einem Land in unterschiedlichen Jahren) das gleiche Grundleiden registriert wird. Dies gilt auch unter der optimistischen Annahme gleicher Verfügbarkeit und Qualität diagnostischer Verfahren, um ein Grundleiden zu Lebzeiten überhaupt feststellen zu können. Damit sind vergleichende Rückschlüsse auf die Bevölkerungsmorbidität anhand der Todesursachenstatistik kaum möglich.

Die Entscheidung, welche Erkrankungen in einem Totenschein eingetragen werden, ist im hohen Maße subjektiv und wird durch landesspezifische Vorlieben beeinflusst, wie es ein deutscher Mediziner in einer privaten Kommunikation einschätzte: „In Deutschland ist es am Ende immer das Herz.“ Anteile von Krebserkrankungen, zerebrovaskulären Erkrankungen (I60–I69) oder Verletzungen als Todesursache unterscheiden sich zwischen Ländern und über die Zeit deutlich weniger.

Es ist aber denkbar, dass auch für andere Erkrankungen, die weniger im Fokus der Gesundheitsberichterstattung und Gesundheitssystemforschung stehen, auffallende Unterschiede zwischen Ländern oder deutliche Veränderungen über die Zeit eher einem unterschiedlichen oder sich wandelnden Grad an Aufmerksamkeit für die Krankheit als tatsächlichen Unterschieden in Morbidität und Gesundheitsversorgung zuzuschreiben sind.

## Todesursachenstatistiken erlauben keine Aussage zur Letalität

Bei der Beschreibung und Interpretation von Mortalitätsraten werden die Begriffe „Sterblichkeit“ und „Mortalität“ häufig synonym für die Mortalitätsrate für eine Erkrankung verwendet. Da der Begriff „Sterblichkeit“ jedoch auch im Sinn von Letalität verstanden wird, kann es zu Fehlinterpretationen der Todesursachenstatistik kommen.

Die *Letalität* einer Erkrankung bezieht sich auf alle Personen, die an der interessierenden Krankheit leiden, und berichtet den Anteil der Verstorbenen aus dieser Gruppe – unabhängig von der registrierten Todesursache (Grundleiden). Die *Mortalität* einer Erkrankung bezieht sich auf die Gesamtpopulation und berichtet den Anteil der Verstorbenen mit der interessierenden Erkrankung als Grundleiden. So gibt die 1‑Jahres-Herzinfarkt-Letalität an, wie groß der Anteil von Personen ist, die innerhalb eines Jahres nach einem erlittenen Herzinfarkt verstarben. Dabei kann dem Tod eines Herzinfarktpatienten auch eine COVID-19-Infektion oder eine Krebserkrankung zugrunde gelegen haben. Die Todesursachenstatistik berichtet dagegen mit der Mortalitätsrate für Herzinfarkt, bei wie vielen Todesfällen anteilig an allen Verstorbenen Herzinfarkt als Grundleiden registriert wurde.

Jedoch schreibt auch das Robert Koch-Institut (RKI) in seiner Gesundheitsberichterstattung: „Es ist daher davon auszugehen, dass Sterberaten und die absolute Anzahl von Todesfällen durch schwere akute respiratorische Infektionskrankheiten auf der Grundlage der Todesursachenstatistik unterschätzt werden, obwohl die Sterblichkeit an ambulant erworbenen, stationär behandelten Pneumonien mit rund 13 % altersabhängig bereits hoch ist“ [30]. In diesem Zusammenhang wird die Letalität von 13 % mit der Mortalitätsrate verglichen, diese Angaben sind aber unabhängig voneinander zu betrachten: Eine hohe Letalität einer Erkrankung bedeutet nicht, dass sich dies in der Todesursachenstatistik 32 widerspiegelt. Dazu müsste die interessierende Erkrankung als Grundleiden qualifiziert sein und eine eineindeutige Beziehung zwischen der Erkrankung und dem Grundleiden bestehen: Erkrankte sterben nur an dieser Erkrankung und bei Angabe der Todesursache lag die Erkrankung sicher vor. Beispielsweise ist bei einer Sepsis zwar die Letalität hoch, die Mortalitätsrate sollte aber gegen null gehen, da es eine intermediäre Todesursache ist und so ggf. die auslösende Infektion als Grundleiden registriert werden müsste.

Ähnlich werden im Herzbericht 2022 die im Vergleich zum Vorjahr gesunkenen Mortalitätsraten für Herzinsuffizienz – eine nichtinformative Todesursache – so erklärt, dass Patienten *mit* Herzinsuffizienz an COVID-19 verstorben sind [17]. Dies wäre weder als Letalität noch als Mortalität zu verstehen. Bei der Interpretation der Mortalitätsraten für Herzinfarkt wird im Herzbericht die „Sterblichkeit“ mit der „Prognose“ zum Jahr davor verglichen. Eine Prognose beschreibt die krankheitsspezifische Letalität, die *nicht* aus Todesursachenstatistiken ableitbar ist.

### Infobox Fallstricke bei der Interpretation der Todesursachenstatistik

- Basis jeder unikausalen Todesursachenstatistik ist *nur* das jeweilige Grundleiden.
- Grundleiden sind Erkrankungen oder externe Ursachen, die am Beginn einer möglichen – zum Tode führenden – Kausalkette stehen und die nicht durch andere Krankheiten oder externe Ursachen ausgelöst wurden.
- Unvollständige oder fehlerhafte Angaben in Totenscheinen beeinträchtigen unmittelbar die Qualität und Validität einer Todesursachenstatistik.
- Eine national und im zeitlichen Verlauf unterschiedlich eingeschätzte Relevanz einzelner Erkrankungen beeinflusst die Mortalitätsraten: Beispielsweise führt eine unterschiedlich häufiger werdende Erfassung von „Demenz“ als Todesursache zu unterschiedlich hohen Mortalitätsraten.
- Wegen hoher Multimorbidität kann eine unikausale Todesursachenstatistik die Morbidität einzelner Grundleiden nur sehr eingeschränkt abbilden.
- Die Morbidität von Erkrankungen, die keine Grundleiden sind, wie Sepsis oder Herzinsuffizienz, kann über die Todesursachenstatistik *nicht* geschätzt werden.
- Mortalitätsraten geben *keine* Auskunft über die Sterblichkeit an einer Krankheit (Letalität) oder über die Prognose.

## Fazit

Die Interpretierbarkeit der Todesursachenstatistik bei vergleichenden Analysen über die Zeit und zwischen Regionen und damit ihr Nutzen für das Gesundheitswesen und die Bevölkerung könnten verbessert werden, wenn die Bedeutung einer sorgfältigen und systematischen Feststellung der Todesursache häufiger kommuniziert würde. Dazu trüge bei, dass eine Leichenschau nicht als ungeliebte Tätigkeit an Assistenzärztinnen und -ärzte delegiert wird, sondern von speziell ausgebildetem und qualifiziertem Personal durchgeführt wird, wie in Finnland und dem Vereinigten Königreich [31]. Regelmäßige Fortbildungen dazu, worauf beim Ausfüllen eines Totenscheins zu achten ist und welche Erkrankungen keine Grundleiden sind, könnten die Qualität der Todesursachenstatistik verbessern [32]. Mit Einführung der elektronischen Todesursachenbescheinigung wären bei besserer Lesbarkeit der Einträge eine Vollständigkeitsprüfung und Vorabplausibilisierung von Kausalketten möglich. Leider befindet sich diese immer noch in der Testphase [33]. Damit könnten die subjektiven, regional und zeitlich divergierenden Einflüsse beim Ausfüllen von Totenscheinen verringert und eine validere Basis für vergleichende Interpretationen des Mortalitätsgeschehens geschaffen werden. Dies ist besonders relevant für die Analyse der kardiovaskulären Mortalität, welche für die Gesundheitssystemforschung und Public Health von speziellem Interesse und eine wichtige Basis für gesundheitspolitische Entscheidungen ist, wie z. B. die aktuell geplante Einrichtung eines neuen Instituts für Prävention durch das Bundesministerium für Gesundheit [34].

Viele der aufgeführten Probleme bei der Nutzung von Todesursachenstatistiken könnten durch die Bereitstellung multikausaler Todesursachendaten vermieden werden. Damit könnte die tatsächliche Morbidität der Bevölkerung abgebildet und valider verglichen werden.

Um bei Ben Jones zu bleiben [1], ist eine in der Mortalitätsstatistik gespeicherte natürliche Todesursache eben *nicht* unbedingt die reelle Todesursache. In der Mortalitätsstatistik wird diejenige Erkrankung als Todesursache eingetragen, die nach einer (unterschiedlich gründlichen) Leichenschau gemäß subjektiver Einschätzung und Präferenzen der leichenschauenden Person – im besten Fall zusammen mit weiteren prävalenten Zuständen und Erkrankungen – in einem Totenschein eingetragen wurde und die anschließend manuell oder automatisiert als Grundleiden selektiert und mittels ICD-10 kodiert wurde.

## Footnotes

1 Medienberichte: z. B. SWR2 Wissen: „Schlampige Leichenschau – Unentdeckte Morde und Suizide“ vom 20.07.2021, MDR Exakt-Die Story: „Ungenaue Leichenschau – wenn Morde unentdeckt bleiben“ vom 12.04.2023.
